# Fission Yeast Methylenetetrahydrofolate Reductase Ensures Mitotic and Meiotic Chromosome Segregation Fidelity

**DOI:** 10.3390/ijms22020639

**Published:** 2021-01-11

**Authors:** Kim Kiat Lim, Hwei Yee Teo, Yuan Yee Tan, Yi Bing Zeng, Ulysses Tsz Fung Lam, Mahesh Choolani, Ee Sin Chen

**Affiliations:** 1 Department of Biochemistry, Yong Loo Lin School of Medicine, National University of Singapore, Singapore 117596, Singapore; bchlimk@nus.edu.sg (K.K.L.); Hwei_Yee_TEO@ttsh.com.sg (H.Y.T.); tanyuanyee@u.nus.edu (Y.Y.T.); yibingzzz@u.nus.edu (Y.B.Z.); lamtszfung@u.nus.edu (U.T.F.L.); 2 National University Health System (NUHS), Singapore 119228, Singapore; obgmac@nus.edu.sg; 3 Department of Obstetrics and Gynaecology, Yong Loo Lin School of Medicine, National University of Singapore, Singapore 119228, Singapore; 4 NUS Graduate School of Integrative Sciences & Engineering, National University of Singapore, Singapore 117456, Singapore

**Keywords:** MTHFR, meiosis, heterochromatin, fission yeast, *Schizosaccharomyces pombe*

## Abstract

Methylenetetrahydrofolate reductase (MTHFR) is a key enzyme in the folate metabolic pathway, and its loss of function through polymorphisms is often associated with human conditions, including cancer, congenital heart disease, and Down syndrome. MTHFR is also required in the maintenance of heterochromatin, a crucial determinant of genomic stability and precise chromosomal segregation. Here, we characterize the function of a fission yeast gene *met11^+^*, which encodes a protein that is highly homologous to the mammalian MTHFR. We show that, although *met11^+^* is not essential for viability, its disruption increases chromosome missegregation and destabilizes constitutive heterochromatic regions at pericentromeric, sub-telomeric and ribosomal DNA (rDNA) loci. Transcriptional silencing at these sites were disrupted, which is accompanied by the reduction in enrichment of histone H3 lysine 9 dimethylation (H3K9me2) and binding of the heterochromatin protein 1 (HP1)-like Swi6. The *met11* null mutant also dominantly disrupts meiotic fidelity, as displayed by reduced sporulation efficiency and defects in proper partitioning of the genetic material during meiosis. Interestingly, the faithful execution of these meiotic processes is synergistically ensured by cooperation among Met11, Rec8, a meiosis-specific cohesin protein, and the shugoshin protein Sgo1, which protects Rec8 from untimely cleavage. Overall, our results suggest a key role for Met11 in maintaining pericentromeric heterochromatin for precise genetic inheritance during mitosis and meiosis.

## 1. Introduction

Eukaryotic DNA is packaged by histones into chromatin, with approximately 146 base pairs (bp) of DNA wrapped around eight histone molecules in a fundamental complex referred to as the nucleosome [1]. Chromatin can be broadly divided into two types: (1) loosely packaged and transcriptionally competent euchromatin, which encompasses most of the genome; and (2) heterochromatin, a transcriptionally silenced and structurally more compact type that shows a low rate of nucleosomal exchange [2,3]. 

Heterochromatin plays an essential role in maintaining genomic stability. In fission yeast, constitutive heterochromatin coats specialized chromosomal regions such as the centromere, sub-telomere, ribosomal DNA, and mating type loci [4]. Pericentromeric heterochromatin forms the foundation for the construction of the kinetochore, connecting the chromosome with spindle microtubules to orchestrate chromosome segregation [5]. Consequently, defects in centromeric heterochromatin often results in lagging chromosomes, owing to unstable microtubule attachment at the centromere [6,7]. Compaction of heterochromatin also serves as a barrier against DNA damage and coats repetitive sequences to prevent the accumulation of non-coding transcripts. Indeed, a loss of heterochromatin integrity improves accessibility of the transcriptional machinery to the DNA, resulting in an increased expression of non-coding transcripts [8,9]. It can also result in an increase in DNA double-stranded break formation [10,11], as a consequence of a disruption to activity of effectors that induce chromatin compaction, for example, histone deacetylases, key enzymatic components of silenced chromatin.

In fission yeast, constitutive heterochromatin requires di- or tri-methylation of the histone H3 lysine 9 (H3K9me2/3), a hallmark of transcriptional silencing or heterochromatin formation, for the maintenance of its integrity [12]. This H3K9me mark recruits factors that contain the chromodomain motif [13], to serve as platforms for recruiting other components with chromatin-modifying activities [2]. Unlike in multicellular eukaryotes, including humans, fission yeast contains only one H3K9 histone methyltransferase.

Methylation reactions involve the transfer of a methyl moiety from the S-adenosyl methionine (SAM) donor onto the lysine or arginine residue of the target protein. SAM is synthesized via the methionine metabolism pathway, which is coupled to the folate cycle [14] that provides metabolites, such as nucleotides and amino acids, for various physiological processes. One of the major enzymes coordinating the folate biosynthesis cycle is methylenetetrahydrofolate reductase (MTHFR). Defects in human MTHFR are proposed to be linked to human aneuploidy, and polymorphisms in MTHFR are correlated with chromosomal non-disjunction in genetic conditions such as Down Syndrome and Turner Syndrome [15,16], even though counter-evidence is also present [17,18]. Furthermore, human MTHFR has been shown to be required for heterochromatin maintenance [19]. Given that the folate and methionine cycles are coupled, the loss-of-function mutation in MTHFR can affect the production of the methyl-group donor SAM, which is essential for a range of methylation reactions, including those on histones [14,20]. The pool of byproducts and metabolites from the folate cycle are employed as substrates and donors for numerous cellular and metabolic processes, including processes that affect chromatin integrity and those that directly regulate the epigenetic inheritance of chromatin states, defects of which underlie the loss of genomic stability within cells [14].

To explore the functional implication of MTHFR in chromosome segregation fidelity, here we sought to investigate the role of a fission yeast MTHFR-related factor, Met11, in regulating genomic stability. In the ∆*met11* null mutant, we observed a significant increase in mitotic chromosome missegregation and hypersensitivity to the microtubule destabilizing drug thiabendazole (TBZ). Consistently, the ∆*met11* mutant showed increased transcription from centromeric heterochromatic regions, signifying defects in chromatin compaction. Heterochromatic derepression was also detected at sub-telomeric and rDNA regions, accompanied by a disruption of H3K9me2 and HP1 protein (Swi6) at all these loci. The ∆*met11* mutation also reduced the precision of meiotic chromosome segregation, which was correlated with a decrease in ascus formation. The meiotic phenotype in the ∆*met11* mutant was synergistically exacerbated in combination with mutations that disrupted sister chromatid cohesion factors. Overall, these results suggest that Met11 functions to maintain centromeric integrity to ensure precise chromosome segregation in mitosis and meiosis.

## 2. Results

### 2.1. Phenotype Analysis of Met11 Disruption Strain

**Figure 1:**
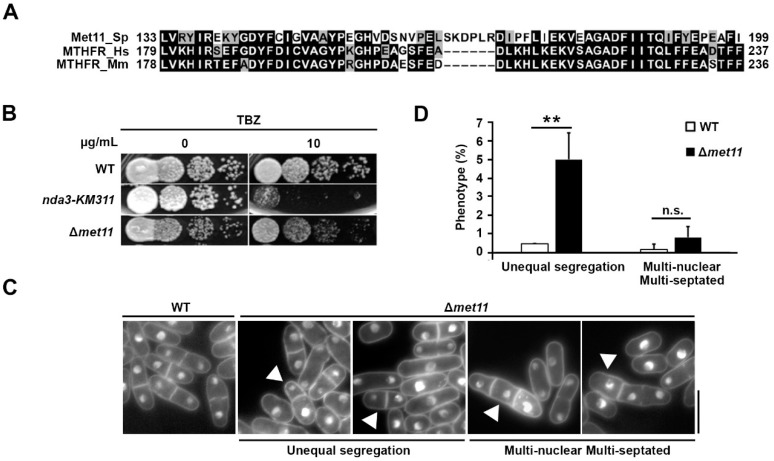
Chromosome missegregation phenotype in loss-of-function *met11* mutant. (**A**) Sequence alignment of fission yeast (*Schizosaccharomyces pombe* (Sp)) Met11 (Met11_Sp) with human (*Homo sapiens* (Hs)) (MTHFR_Hs) and mouse (*Mus musculus* (Mm)) MTHFR (MTHFR_Mm) homologs. Black, same amino acids; grey, similar amino acids. (**B**) Spot test of serially diluted WT, *nda3-KM311* and ∆*met11* strains on media untreated or treated with 10 µg/mL thiabendazole (TBZ). *nda3-KM311*, tubulin beta Nda3 gene as positive control. (**C**) Morphology of log-phase WT and ∆*met11* cells. Arrow is unequal chromosome segregation or multinucleated-multiseptated cells. Bar: 10 µm. (**D**) Proportion of cells exhibiting unequal chromosome segregation and multinucleated/multiseptated phenotypes in ∆*met11* and WT cells. *n* ≥ 200 cells with >1 nucleus. Bars and error bars represent mean ± S.D. respectively obtained from three experiments. n.s., not significant; **: *p* < 0.01.

The fission yeast genome consists of two genes that encode MTHFR-like proteins: *met9* and *met11* [21]. Comparing the protein sequences, we noted high similarity among human and murine MTHFR and fission yeast Met11 (Figure 1A). To explore the roles of these two proteins, we first constructed *met9* and *met11* mutants by replacing the coding sequence with the G418-resistance cassette and *ura4^+^* gene [22,23,24]. However, we were only able to generate the ∆*met11* mutant, which is consistent with a recent genome-wide gene deletion analysis that showed that *met9* is essential for growth [25]. 

Next, we tested the growth of ∆*met11* cells in the presence of several cytotoxic agents. Among these, ∆*met11* showed a low but reproducible susceptibility to the microtubule destabilizing drug, TBZ (Figure 1B), but not to hydroxyurea (HU), camptothecin (CPT), and methyl methanesulfonate (MMS) (Supplementary Figure S1). Susceptibility to TBZ is often correlated with defects in the execution of mitotic chromosomal segregation [22,26]. To check whether defects in Met11 were connected to aneuploidy in fission yeast, we studied the nuclear segregation phenotype of the ∆*met11* mutant using microscopy (Figure 1C). Although the distribution of cells at various stages of the cell cycle was largely indistinguishable from that of wild-type (WT) cells (refer below), we observed a 5.0 ± 0.8% upregulation in ∆*met11* cells exhibiting unequal nuclear division phenotype (Figure 1C,D). A marginal increase in multi-septated and multi-nucleated cells was also detected (Figure 1C,D). 

### 2.2. Pericentromeric Heterochromatin Defects in Met11 Null Mutant

**Figure 2:**
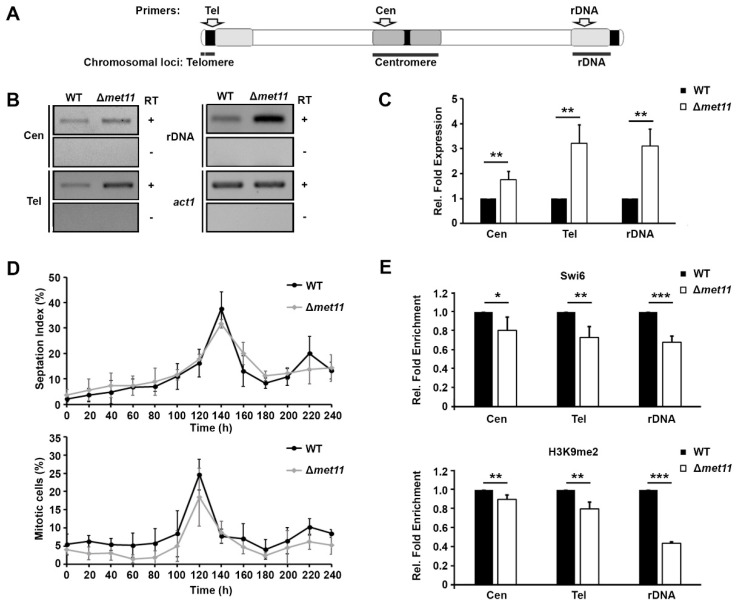
Deletion of *met11* disrupts heterochromatic integrity at pericentromeric, sub-telomeric and ribosomal DNA loci. (**A**) Schematic diagram to show the chromosomal location of constitutive heterochromatic loci and the location of the primers used in the study. (**B**) Detection of transcripts derived from centromeric (Cen), sub-telomeric (Tel), and ribosomal DNA (rDNA) heterochromatic loci of asynchronous WT and Δ*met11* cells using RT-PCR (RT). +, RT-PCR reaction with active reverse transcriptase; -, RT-PCR reaction with inactivated reverse transcriptase. *act1*, actin gene as loading control. Results shown are representative of three independent experiments. (**C**) Quantification of levels of heterochromatic transcripts in ∆*met11* relative to WT and normalized to *act1* transcription in (**B**). Bars and error bars represent mean ± S.D. respectively obtained from three experiments. **: *p* < 0.01. (**D**) Percentage of 2 N cells with (septation index) or without septum (mitotic cells) of HU block-release-synchronized *Δmet11* and WT cells. *n* ≥ 200. Bars and error bars represent mean ± S.D., respectively from three experiments. (**E**) Chromatin immunoprecipitation analyses of Swi6 and H3K9me2 levels associated with heterochromatic sequences at centromeric (Cen), sub-telomeric (Tel) and ribosomal DNA (rDNA) regions in Δ*met11* relative to WT and normalized to *act1* signals using quantitative (q)-PCR. Bars and error bars represent mean ± S.D. respectively obtained from three independent experiments. *: *p* < 0.05, **: *p* < 0.01, ***: *p* < 0.001.

The chromosome missegregation phenotype of ∆*met11* suggested the possibility of defects in centromeric chromatin integrity. Fission yeast centromeres comprise stretches of heterochromatized repeat sequences that flank a region of specialized chromatin containing the centromere-specific histone H3 variant CENP-A [27] (Figure 2A). Centromeric heterochromatin is transcriptionally silent except during a brief window when the cells are in S-phase [9]. Thus, we used RT-PCR to measure changes in transcription in the ∆*met11* and WT cells. We found an approximately 1.8-fold (±0.3) upregulation of centromeric repetitive sequences in ∆*met11* relative to WT (Cen, Figure 2B,C), and also noted 3.2-fold (±0.8) and 3.1-fold (±0.7), respectively, from blocks of constitutive heterochromatin at the sub-telomeric region (Tel) and ribosomal DNA sequences (rDNA) (Figure 2B,C), in ∆*met11* compared to WT. 

Non-coding transcription at major heterochromatic loci is upregulated during a brief period within S-phase [9,28]. To check whether the upregulation of heterochromatic transcript observed in ∆*met11* cells may be attributable to a prolonged S-phase, we synchronized WT and ∆*met11* cells from G1/S-phase by blocking the cell cycle with HU and releasing for sampling at regular time intervals (20 min). We followed the proportions of cells with division septa (septation index), and bi-nucleated cells without septa to define mid-late S and late-M (anaphase)-G1/S phases of the cell cycle, respectively [9,29]. The complete overlap of the cell cycle profiles between ∆*met11* with WT cell cycle demonstrated that ∆*met11* was not aberrantly delay in S-phase (Figure 2D). To further ascertain that heterochromatin was indeed disrupted, we measured the levels of H3K9me2 and Swi6 at heterochromatic domains using chromatin immunoprecipitation (ChIP). We detected a 19.6%, 26.9%, and 32.1% reduction in Swi6 binding (Figure 2E, top); as well as 10.0%, 19.8%, and 56.2% decrease in H3K9me2 levels at Cen, Tel, and rDNA, respectively (Figure 2E, bottom). Taken together, these results indicate that Met11 is required to maintain the integrity of constitutive heterochromatin at peri-centromeric, sub-telomeric and rDNA regions, and suggest that defects associated with centromeric heterochromatin may cause the chromosomal missegregation phenotype observed in ∆*met11* cells. 

### 2.3. Sporulation Defect in Met11 Null Mutant

**Figure 3:**
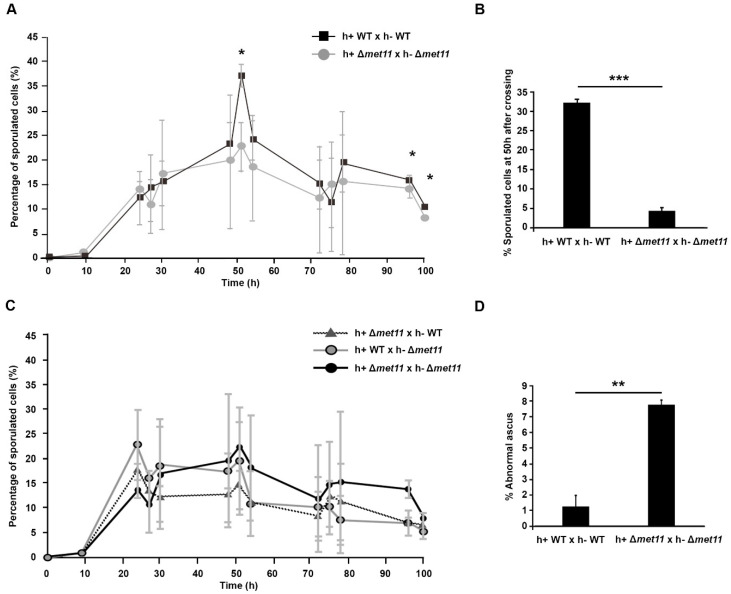
Meiotic defects in the absence of *met11* function. (**A**) Proportion of cells undergoing sporulation following h^+^ WT × h^−^ WT (black) and h^+^ ∆*met11* × h^−^ ∆*met11* (grey) mating during 100 h. *n* > 1000; *: *p* < 0.05. (**B**) Repeat of (**A**) at 50 h after mating of cells. Error bars: S.D. from three experiments. ***: *p <* 0.001. (**C**) Sporulation frequency in mating cultures of h^+^ ∆*met11* × h^−^ ∆*met11* (black), h^+^ WT × h^−^ ∆*met11* (grey), and h^+^ ∆*met11* × h^−^ WT (dotted line) during 100 h after mating. *n* > 1000. Mean values are from five experiments. Error bars: S.D. (**D**) Graph shown the mean of ascus with chromosome segregation defects in h^+^ WT × h^−^ WT and h^+^ ∆*met11* × h^−^ ∆*met11.* Error bars: S.D. from three experiments. **: *p <* 0.01.

MTHFR polymorphisms are connected to birth defects and defects during embryonic growth in mammals, and this may suggest reduced meiotic chromosome segregation fidelity in ∆*met11* cells. To test this, we next measured changes in sporulation in ∆*met11* cells relative to WT cells. Opposite mating types of WT and ∆*met11* cells were mated on nitrogen-depleted media, and the proportion of sporulating cells were documented by counting spore-containing asci under light microscopy over 100 h (Figure 3A). The sporulation frequency peaked at 50 h in both WT and ∆*met11* (Figure 3A); albeit sporulation was 7.3-fold much lower in the ∆*met11* cells (Figure 3B). We then tested whether ∆*met11* reduced sporulation in a dominant or recessive manner by preparing crosses of the ∆*met11* strain with WT cells using separate mating types of ∆*met11* cells in two heterozygous crosses relative to homozygous ∆*met11* crosses. Over the 100 h, we were unable to detect significant differences in sporulation efficiency between the homozygous or heterozygous crosses of ∆*met11* and noted no spike in sporulation at 50 h (Figure 3C). Taken together, these observations suggest that ∆*met11* dominantly acted to affect sporulation and meiosis.

We noticed some asci in ∆*met11* crosses contained aberrant spore number. To study these defects further, we ethanol-fixed asci of h^+^ WT × h^−^ WT and h^+^ ∆*met11* × h^−^ ∆*met11* for staining with DAPI at 50 h after mating. At this time point, approximately 7.8 ± 1.9% of cells contained only three spores instead of four. Similar aberrant asci were seldom observed in the WT cells (Figure 3D). Staining of the nucleus by DAPI revealed three nuclei instead of four in these abnormal asci (Supplementary Figure S2). 

### 2.4. Met11 Synergistically Govern Meiosis Meiotic Fidelity with Shugosin and Cohesin 

**Figure 4:**
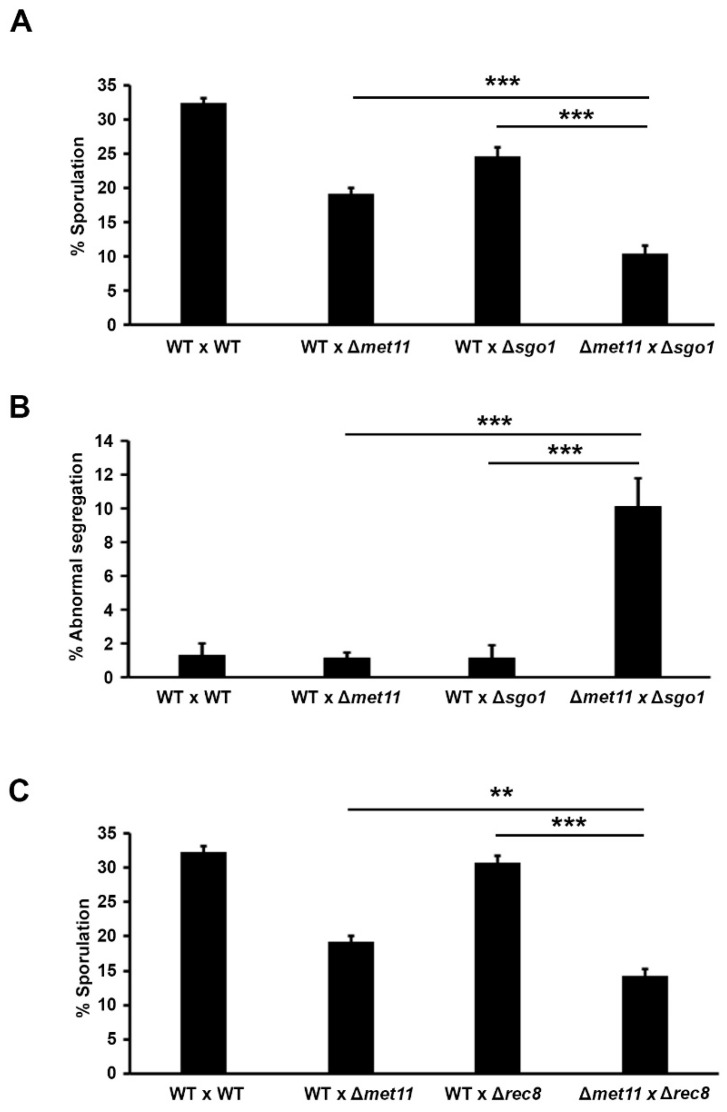
Met11 synergistically interacts with shugoshin and cohesin to govern proper meiosis. (**A**) Ascus formation frequency of WT × WT, WT × ∆*met11*, WT × ∆*sgo1,* and ∆*met11* × ∆*sgo1* at 50 h after mating. *n* > 200. The result is the mean of three experiments. Error bar: S.D. ***: *p* < 0.001. (**B**) Genetic interaction of Met11 and Sgo1 on chromosome missegregation. Proportion of ascus containing abnormal (non-tetrad) number of nuclei was counted at 50 h after mating by microscopic observation of DAPI-stained cells. Crosses were WT × WT, WT × ∆*met11*, WT × ∆*sgo1*, and ∆*met11* × ∆*sgo1*. *n* > 200. Result represent mean of three experiments. ***: *p* < 0.001. (**C**) Ascus formation frequency of WT × WT, WT × ∆*met11*, WT × ∆*rec8* and ∆*met11* × ∆*rec8* at 50 h after mating. *n* > 200. Bars and error bars represent the mean and S.D. of three experiments. **: *p* < 0.01, ***: *p* < 0.001.

The timely cleavage of the centromeric cohesin complex is key for the reduction division that yields the haploid complement of chromosomes in meiosis [30]. Defects in the meiosis-specific cohesin complex subunit Rec8 and the Rec8-protector shugoshin (Sgo1 in fission yeast) cause a profuse meiotic chromosome segregation defect [31]. Since Sgo1 and Rec8 localize to centromeric heterochromatin, we tested whether Met11 was epistatic with Sgo1. We crossed ∆*met11* and ∆*sgo1* (Figure 4A,B) and quantified the frequency of ascus formation in WT, single (∆*met11* and ∆*sgo1*), and double mutants (∆*met11*∆*sgo1*). The WT × ∆*met11* cross showed 19.2 ± 0.9% sporulation, which was lower to that in WT × WT cross (32.4 ± 0.8%). Sporulation in the WT × ∆*sgo1* cross was about 1.3-fold lower, with 24.6 ± 1.3% asci formation. Sporulation efficiency, however, was further synergistically abolished in the ∆*met11* × ∆*sgo1* cross, at 10.4 ± 1.1% (Figure 4A). In contrast, the chromosome missegregation defect in the asci was highest in the ∆*met11* × ∆*sgo1* cross as compared with the other three crosses (Figure 4B). Overall, these findings suggest that Met11 and Sgo1 act in functionally parallel pathways in the execution of meiosis in fission yeast.

To further confirm the genetic relationship between Met11 and sister chromatid cohesion-associated mechanisms in regulating meiosis, we next investigated the genetic interaction between *met11* and *rec8*. WT × WT, WT × ∆*met11*, and WT × ∆*rec8* resulted in 32.4 ± 0.8%, 19.2 ± 0.9%, and 30.8 ± 1.0% sporulation, respectively, with cumulative reduction in sporulation for the ∆*met11* × ∆*rec8* cross at 14.3 ± 0.9% (Figure 4C). Thus, Met11 and Rec8 may similarly act in parallel to govern proper meiotic progression. 

## 3. Discussion

The connection between MTHFR and aneuploidy in human non-disjunction syndrome is controversial. In this study, we explored the role of the fission yeast MTHFR, Met11, on chromosome segregation. We found that a loss of Met11 inhibited precise chromosome segregation and was associated with a disruption in centromeric heterochromatin. Overall, our analyses revealed a role for Met11 in governing proper meiotic progression in conjunction with shugoshin and cohesin (Supplementary Figure S3). 

Meiotic chromosome segregation occurs in two stages to produce four haploid meiotic progenies, and the co-segregation of homologous chromosomes during meiosis I is mediated through the protection of centromeric cohesin from cleavage by the shugoshin protein. This protection is a key step to ensure the precise segregation in meiosis I by permitting mono-orientation of sister kinetochores to be captured by spindle microtubules from the same pole of the cell [32]. In fission yeast, the recruitment of shugoshin to protect cohesin at the centromere is associated with heterochromatin [33], and this close association between heterochromatin and cohesin predicts that Met11 and Sgo1/Rec8 would be epistatic. However, our findings showed otherwise, with the concurrent disruption of *met11* and *sgo1*/*rec8* causing a prominent cumulative increase in meiotic defects. One possibility to the unexpected genetic interaction may arise from the multiple regulatory pathways that localize Sgo1 to centromere. In addition to binding directly to Swi6, Sgo1 is recruited via phosphorylation of histone H2A by the checkpoint kinase Bub1 [33,34]. Centromeric accumulation of Bub1 is dependent on polo-like kinase Plo1 that is recruited by Moa1/Meikin, but Moa1-Plo1 appears to also regulate Sgo1 localization independently of Bub1 [35]. On the other hand, it is also possible that Sgo1 and Rec8 may have roles that are functionally distinct from that of sister chromatid cohesion that is regulated in conjunction with heterochromatin during meiotic chromosome segregation. In fact, Shugoshin was reported to regulate meiotic prophase checkpoint in worm [36] and promote kinetochore microtubule stability [37]. Rec8 plays additional role to organize meiotic chromosome organization and modulate chromosomal compaction [38,39]. Furthermore, Rec8, especially that binding on chromosome arms facilitate meiotic recombination in conjunction with other recombination regulators such as Rec11 and Rec12 [38,40]. Alternatively, Met11 may also function independently of heterochromatin to regulate meiosis in a parallel manner relative to Sgo1/Rec8, for example, supplying nucleotides for DNA synthesis via the methionine-folate metabolic cycles [14]. 

MTHFR affects centromeric heterochromatin integrity in fission yeast is consistent with the observation from human [41,42]. However, a new finding illuminated by our data is the function of Met11 to also maintain heterochromatin integrity at the sub-telomeric and rDNA loci. Although the mechanistic implication of this relationship is unclear, it is possible that MTHFR may act to maintain heterochromatic compaction in these regions. Fission yeast Swi6 has been shown to counteract the recruitment of meiotic recombination protein Rec10 so as to prevent forming double-stranded DNA breaks and crossing-over for sister chromatid exchange at peri-centromeric heterochromatin [43]. Similar mechanism may likely operate on the sub-telomeric and rDNA heterochromatic loci. These regions are enriched with repetitive sequences that can easily undergo recombination and if not properly repaired, can result in loss of genomic stability. Incomplete resolution of recombination products between repetitive sub-telomeric and rDNA sequences of juxtaposed chromosomes could possibly arise from the weakening of heterochromatin in the absence of *met11.* The resulting linkages may in turn give rise to the chromosomal non-disjunction phenotypes observed during chromosomal segregation in Δ*met11* cells. 

Polymorphisms in the MTHFR gene have been implicated in many human diseases including congenital heart disease, cancers, infertility, and hereditary syndromes [14,44,45,46]. However, multiple population-based studies performed across different geographical locations have yet to yield a consensus on the potential connections among the various MTHFR polymorphisms. For example, MTHFR polymorphisms have been associated [47,48] and not associated [49,50] with Down syndrome. However, an extensive literature search for polymorphisms associated with Down syndrome indicates a trend toward phenotypic penetrance that is dependent on the co-occurrence of two or more polymorphisms [14]. Although polymorphisms in shugoshin and cohesin have not been reported to be associated with genetic disorders like Down syndrome, the genetic interaction observed here suggests that polymorphisms in genes governing sister chromatid separation in meiosis may only manifest alongside other polymorphisms, such as those in MTHFR. 

In conclusion, our work characterized the MTHFR-homologous gene *met11* of fission yeast and showed that it regulates pericentromeric integrity and meiotic chromosome segregation. A genetic interaction exists between *met11* and *sgo1/rec8* in the regulation of meiotic chromosome segregation and sporulation. Similar interactions will be explored in future studies to understand the genetic basis for aneuploidies, such as Down syndrome.

## 4. Materials and Methods 

### 4.1. Fission Yeast Manipulation

Fission yeast techniques were followed as previously described [22,51], with strains cultured in YEA media (0.5% yeast extract (BD Biosciences, Franklin Lakes, NJ, USA), 75 mg/L adenine, 3% d-glucose (Sigma-Aldrich, St Louis, MO, USA)). The ∆*met11* mutant was constructed by replacing the coding locus with a kanamycin-resistant gene cassette in the h^−^ 972 prototrophic WT strain or *ura4* gene cassette in uracil auxotrophic strain [23]. Disruption was confirmed using PCR with locus-specific primers. Strains of different mating type and varied genetic background were obtained by standard mating and tetrad dissection procedures using an MSM micromanipulator (Singer Instruments, Watchet, Somerset, UK). Cell synchronization was performed by arresting the cells with 15mM hydroxyurea (HU) for 3 h then releasing upon washing away the drug. Cells were collected at 20 min intervals.

### 4.2. Spotting Assay

Asynchronous log-phase cells were 10-fold serially diluted and spotted onto solid YEA media containing 2% Bacto agar (BD Biosciences, Franklin Lakes, NJ, USA) with or without drugs. Growth was documented after incubation at 30 °C for 3 to 4 days, as previously described [52,53].

### 4.3. Microscopy

Log-phase mitotic cells were fixed with 10% glutaraldehyde (Sigma-Aldrich, St Louis, MO, USA) and stained with 50 µg/mL 4′,6-diamidino-2-phenylindole (DAPI) (Thermo Fisher Scientific, Waltham, MA, USA) before observation under Nikon Eclipse Ti-E fluorescence microscope (Nikon, Tokyo, Japan), as previously described [22,54]. Meiotic cells in asci were fixed with 70% ethanol for 30 min and washed three times with phosphate saline buffer (PBS). Over 200 DAPI-stained asci were counted to quantify chromosome segregation fidelity.

### 4.4. Sporulation Frequency Quantification

Cells of opposite mating types were mated at 26 °C on sporulation agar (SPA) (1% d-glucose, 0.1% KH_2_PO_4_, 0.1% of 1000× vitamin (4.2 mM pantothenic acid, 81.2 mM nicotinic acid, 55.5 mM inositol, 40.8 mM biotin), and 0.0045% of each of adenine, histidine, leucine, uracil, lysine hydrochloride, pH 5.5 in 2% Bacto agar) (Sigma-Aldrich, St Louis, MO, USA). Cells were taken from the mating mixture at regular time points over 4 days to quantify the proportion of cells (*n* > 2000) that had undergone sporulation. Counting was performed using an Olympus CX31 light microscope (Olympus Corporation, Tokyo, Japan). The number of asci containing four spores over the total number of cells counted was expressed as % in Figure 3 and Figure 4. Ascus containing less than four spores were scored as abnormal. 

### 4.5. Reverse Transcriptase Polymerase Chain Reaction (RT-PCR)

Total RNA was extracted from log-phase cells with TRIzol (Thermo Fisher Scientific, Waltham, MA, USA), treated with DNase I (New England Biolabs, Ipswich, MA, USA), and extracted with phenol:chloroform:isoamyl alcohol (25:24:1) (Nacalai Tesque, Kyoto, Japan). RNA (100 ng) was then used for one-step RT-PCR using a OneStep RT-PCR kit (Qiagen, Venol, Netherlands), as previously described [22,54]. The primers sequence used for reverse transcription and subsequent PCR steps were as follows. For pericentromere (Cen): 5′ GAAAACACATCGTTGTCTTCAGAG 3′ and 5′ CGTCTTGTAGCTGCATGTGAA 3′. For sub-telomere (Tel): 5′ CAACACCAATACTGACGATGATG 3′ and 5′ GCAATAGAACCAGCGGTTTG 3′. For rDNA region (rDNA): 5′ CACTTGAGCCTCATGATGTGTTC 3′ and 5′ ATGGAGAAGGCTGGAATGCA 3′. For the loading control actin (*act1*): 5′ GGCATCACACTTTCTACAACG 3′ and 5′ GAGTCCAAGACGATACCAGTG 3′. PCR products were resolved in agarose gel. Bands intensity were quantified using ImageQuant TL software ver 8.1 (GE Healthcare, Little Chalfont, UK). Relative fold expression of mutant over wild-type cells were calculated by comparing the expression of target sequence over loading control *act1*. 

### 4.6. Chromatin Immunoprecipitation (ChIP)

Previously published procedures were followed closely [55,56]. Briefly, log-phase growing cultures (OD_600nm_ = 0.5) were fixed with 3% paraformaldehyde (Sigma-Aldrich, St Louis, MO, USA) before homogenization with glass beads. Cell extracts were then sonicated to shear the DNA followed by immunoprecipitation using Swi6 and H3K9me2 antibodies (ab188276 and ab1220, respectively, Abcam, Cambridge, UK). Reverse crosslink was achieved at 65 °C overnight, and treated with proteinase K (Thermo Fisher Scientific, Waltham, MA, USA). Ethanol precipitated DNA was treated with RNase A (Roche Diagnostic, Basel, Switzerland) before qPCR was done using the same Cen, Tel and rDNA primers as stated for RT-PCR. 

### 4.7. Quantitative PCR (qPCR)

ChIP samples were subjected to qPCR using iTaq Universal SYBR Green Supermix (Bio-Rad, Hercules, CA, USA) with amplification and detection by StepOne real time PCR system (Applied Biosystems, Foster City, CA, USA). Primers used for targeting the different heterochromatic region such as Cen, Tel, rDNA and *act1* were the same as those used in RT-PCR. The fold enrichment of immunoprecipitated DNA relative to total input DNA (whole cell extract) was determined using ΔΔCt method in which *act1* was used as internal control. Further analysis standardized the mutant results with those of wild-type strain to obtain a relative fold enrichment.

### 4.8. Statistical Data Analysis

Unpaired Student’s *t*-test was used to determine the significance of difference among different samples tested in Microsoft Excel (Microsoft Corporation, Redmon, WA, USA). At least three independent repeats were performed for all experiments. Sample size (*n*) and replicates were mentioned in the figure legend. *p* values < 0.05, <0.01 and <0.001 were represented by asterisk *, ** and ***, respectively.

## Data Availability

Not applicable.

## Supplementary Materials

Supplementary materials can be found at https://www.mdpi.com/1422-0067/22/2/639/s1.

## Abbreviations

MTHFR	Methylenetetrahydrofolate reductase
bp	Base pair
H3K9me2/3	Di- or tri-methylation of histone H3 lysine 9
SAM	S-adenosyl methionine
TBZ	Thiabendazole
WT	Wild-type
Cen	Centromere
rDNA	Ribosomal DNA
Tel	Telomere
h	hour
Sp	*Schizosaccharomyces pombe*
Hs	*Homo sapiens*
Ms	*Mus musculus*
RT-PCR	Reverse transcriptase polymerase chain reaction
DAPI	4′,6-diamidino-2-phenylindole
SPA	Sporulation agar
