# Supplementary material for: Fission Yeast Methylenetetrahydrofolate Reductase Ensures Mitotic and Meiotic Chromosome Segregation Fidelity
Source: Int J Mol Sci. 2021 Jan 11;22(2):639. doi: 10.3390/ijms22020639 (PMC7827777; doi:10.3390/ijms22020639)
Supplement: Supplementary file 1 [file ijms-22-00639-s001.pdf]

Supplementary Figures

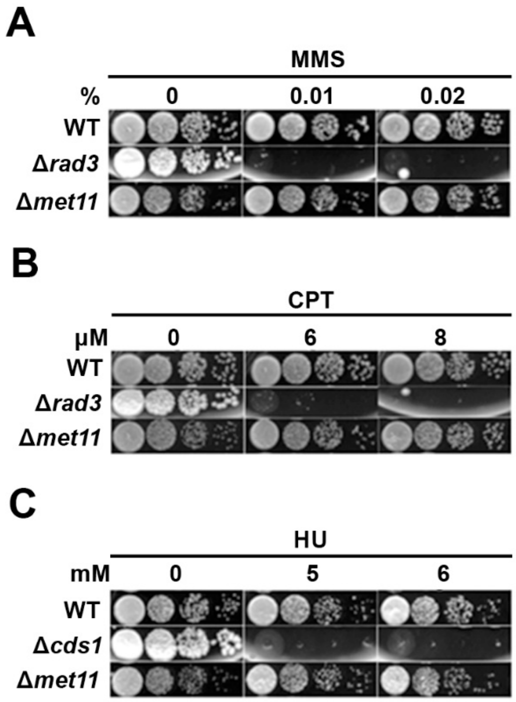

Figure S1. Loss of *met11* does not affect tolerance to cytotoxic agents: (**A**) methyl methanesulfonate (MMS), (**B**) hydroxyurea (HU), or (**C**) camptothecin (CPT). Δ*rad3* and Δ*cds1* were used as control for respective cytotoxic agents.

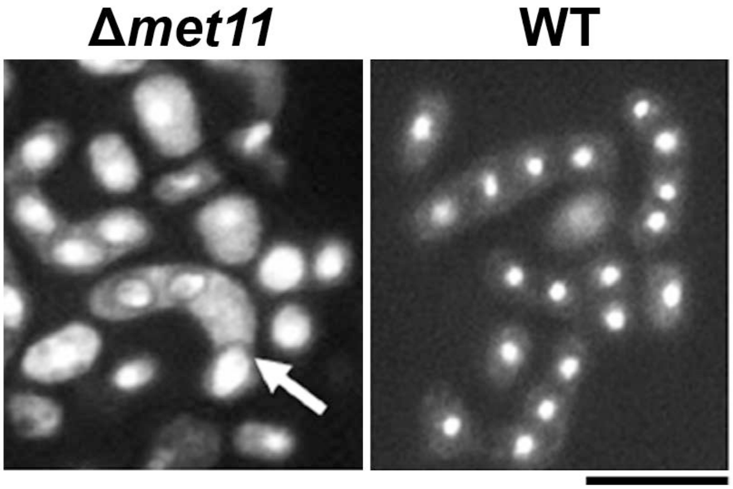

Figure S2. Loss-of-function *met11* mutant shows ascus with an aberrant number of nuclei. Samples were fixed and stained with DAPI for microscopy. Arrow: ascus with aberrant number of nuclei. Left, Δ*met11*; Right, WT. Bar: 10 μm.

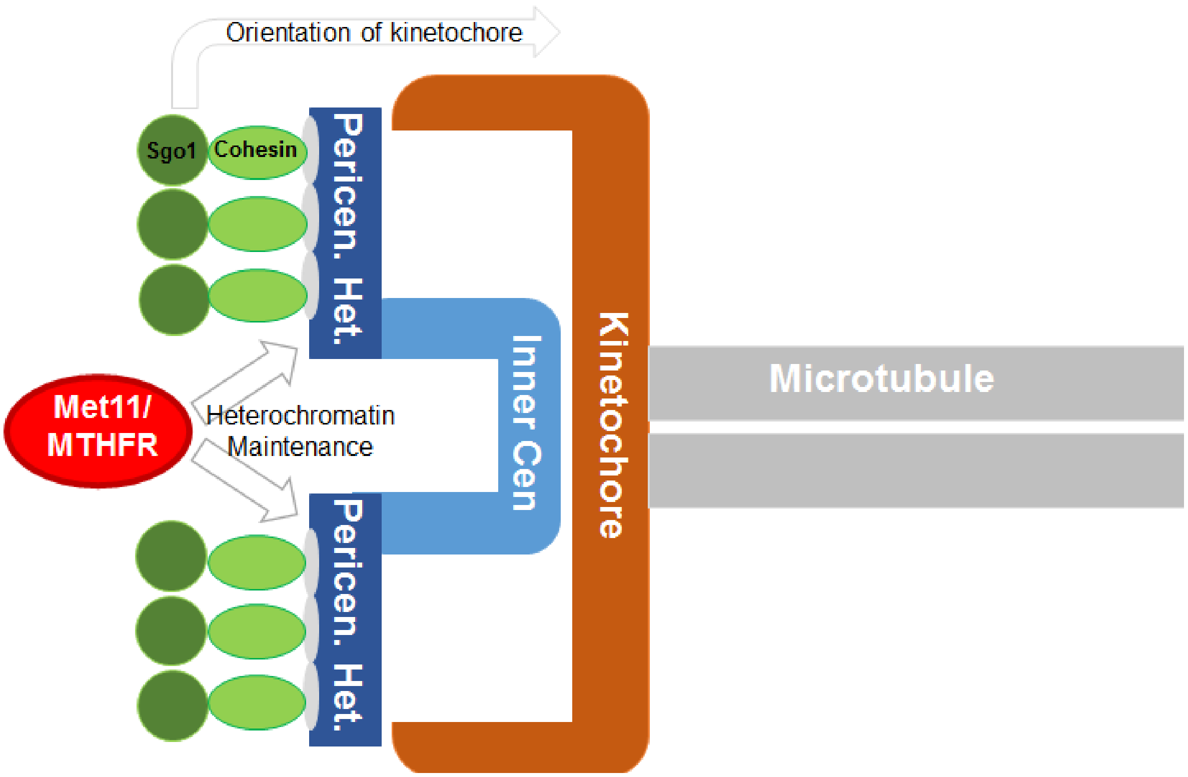

Figure S3. Schematic representation of the action of Met11 in cooperation with cohesin/shugoshin via composite modulation on the kinetochore and the underlying chromatin at the centromere. Cen: centromere; Pericen. Het.: pericentromeric heterochromatin.
